# Weight loss in adult male Wistar rats by Roux-en-Y gastric bypass is primarily explained by caloric intake reduction and presurgery body weight

**DOI:** 10.1152/ajpregu.00169.2023

**Published:** 2024-04-08

**Authors:** C. Warner Hoornenborg, Edit Somogyi, Jan E. Bruggink, Christina N. Boyle, Thomas A. Lutz, Marloes Emous, André P. van Beek, Csaba Nyakas, Gertjan van Dijk

**Affiliations:** ^1^Department of Behavioral Neuroscience, Groningen Institute for Evolutionary Life Sciences (GELIFES), University of Groningen, Groningen, The Netherlands; ^2^Department of Endocrinology, University Medical Center Groningen, Groningen, The Netherlands; ^3^School of PhD Studies, University of Physical Education, Budapest, Hungary; ^4^Institute of Veterinary Physiology, Vetsuisse Faculty University of Zurich, Zurich, Switzerland; ^5^Department of Bariatric and Metabolic Surgery, Medical Center Leeuwarden, Leeuwarden, The Netherlands

**Keywords:** bariatric surgery, energy balance, physical activity, weight loss

## Abstract

Diets varying in macronutrient composition, energy density, and/or palatability may cause differences in outcome of bariatric surgery. In the present study, rats feeding a healthy low-fat (LF) diet or an obesogenic high-fat/sucrose diet (HF/S) were either subjected to Roux-en-Y gastric bypass surgery (RYGB) or sham surgery, and weight loss trajectories and various energy balance parameters were assessed. Before RYGB, rats eating an HF/S (*n* = 14) diet increased body weight relative to rats eating an LF diet (*n* = 20; *P* < 0.01). After RYGB, absolute weight loss was larger in HF/S (*n* = 6) relative to LF feeding (*n* = 6) rats, and this was associated with reduced cumulative energy intake (EI; *P* < 0.05) and increased locomotor activity (LA; *P* < 0.05–0.001), finally leading to similar levels of reduced body fat content in HF/S and LF rats 3 wk after surgery. Regression analysis revealed that variation in RYGB-induced body weight loss was best explained by models including *1*) postoperative cumulative EI and preoperative body weight (*R*^2^ = 0.87) and *2*) postoperative cumulative EI and diet (*R*^2^ = 0.79), each without significant contribution of LA. Particularly rats on the LF diet became transiently more hypothermic and circadianally arrhythmic following RYGB (i.e., indicators of surgery-associated malaise) than HF/S feeding rats. Our data suggest that relative to feeding an LF diet, continued feeding an HF/S diet does not negatively impact recovery from RYGB surgery, yet it promotes RYGB-induced weight loss. The RYGB-induced weight loss is primarily explained by reduced cumulative EI and higher preoperative body weight, leading to comparably low levels of body fat content in HF/S and LF feeding rats.

**NEW & NOTEWORTHY** Relative to feeding an LF diet, continued feeding an HF/S diet does not negatively impact recovery from RYGB surgery in rats. Relative to feeding an LF diet, continued feeding an HF/S diet promotes RYGB-induced weight loss. The RYGB-induced weight loss is primarily explained by reduced cumulative EI and higher preoperative body weight, leading to comparably low levels of body fat content in HF/S and LF feeding rats.

## INTRODUCTION

Bariatric surgery has been proven to produce successful long-term weight loss and to reduce incidences of type 2 diabetes mellitus (1), heart disease, and cancer (2). In the past decades, Roux-en-Y gastric bypass (RYGB) surgery has become the gold standard method to produce these effects (3) and successfully reduce mortality and morbidity rates (4, 5).

Despite this excellent track record, some of the underlying mechanisms of weight loss after RYGB surgery are still not clearly understood. Given that obesity is recognized as a chronic and relapsing condition, a long-term treatment plan for contributing factors to optimize weight loss postoperatively is often needed (6). For example, variation in eating behavior following bariatric surgery is a factor causing rather large interindividual differences in weight loss (7). In preclinical studies, animals that have undergone RYGB surgery exhibit a decreased preference for high-carbohydrate (8) and high-fat foods (9, 10), which could support weight loss. It is, however, still unclear whether the altered food preference affects energy intake per se (11), as another report showed that early postoperative exposure to high-fat food did not lead to fat avoidance nor superior weight loss in the long-term after RYGB surgery (12). Rats in fact showed avoidance toward a high-fat diet following RYGB surgery (8, 13); however, no clear alterations are seen in clinical studies on food preferences (14).

Another predictor of weight loss after bariatric surgery is physical activity during the early recovery phase after surgery (15). It may be difficult, however, to adhere to the recommended physical activity levels (15, 16), particularly if voluntary activity would be negatively influenced by bariatric surgery. A third factor that may influence weight loss is the fat reserves present before bariatric surgery. A study by Hao et al. (17) showed that RYGB-induced weight loss in mice was proportional to initial fat content and body weight, and this was also observed in some clinical studies (18, 19).

In the present study, we investigated some of the abovementioned factors in weight loss following RYGB in rats. To this end, rats were subjected to a low-fat, fiber-rich laboratory diet (LF) or switched to a highly palatable and obesogenic high-fat diet enriched with sucrose (HF/S) 3 wk before RYGB and followed their weight loss trajectories, energy intake, and voluntary locomotor patterns after RYGB. Based on earlier work (18, 19), we hypothesized that the rats on the obesogenic HF/S would lose more weight following RYGB (because they were anticipated to be heavier and have more body fat). Another hypothesis could be that the rats on the HF/S diet would show less body weight and fat loss after RYGB, because of the higher palatability of the HF/S diet relative to the LF diet (20).

Alternatively, body weight loss could be augmented in rats eating an HF/S diet due to the fact that rats do not prefer the diet (20, 21). Besides that, diets high in fat have been shown to induce inflammation and metabolic derangements (22, 23), which may aggravate surgery-induced malaise resulting in reduced energy intake as well, potentially explaining postoperative differences between rats eating an LF and HF/S diet. For this purpose, we also assessed, besides locomotor activity, circadian fluctuations in body temperature by continuous telemetric recordings, allowing us to assess how well the diets were tolerated in the face of RYGB surgery.

## MATERIALS AND METHODS

### Animals

All animal procedures have been conducted according to relevant national and international guidelines and were approved by the Animal Experimentation Committee of the University of Groningen. Adult male Wistar rats (*n* = 34; weighing 483 ± 8) obtained from Harlan were individually housed in Plexiglas cages (25 × 25 × 30 cm) on a layer of wood shavings with a gnawing stick. Room temperature was set at 20 ± 2°C, humidity was set at 60 ± 5%, and the light/dark cycle was 12:12 h (lights ON at 09:00 AM). Animals were handled daily and weighed before lights were off. Water and standard chow were provided ad libitum.

### Overall Experimental Design

**Figure 1.**
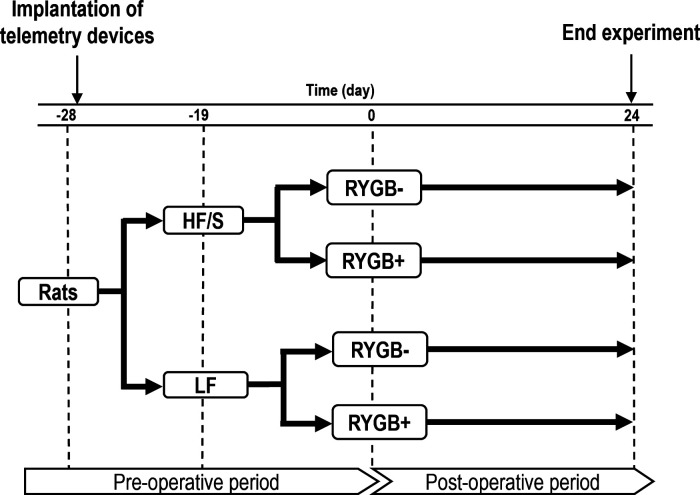
Experimental design and timeline of the experiment. The experiment is divided by the period before Roux-en-Y gastric bypass (RYGB+) or sham surgery (RYGB−; indicated as preoperative) and the period after RYGB+/RYGB− (indicated as postoperative). HF/S, high fat with sucrose diet; LF, low-fat diet.

Twenty-eight days before RYGB surgery (RYGB+) or sham surgery (RYGB−), rats were weighed on the basis of which four groups were established with comparable body weights, namely, RYGB+ (*n* = 12) and RYGB− (*n* = 8) that were maintained on feeding the regular low fat (LF) throughout the entire experiment. The other two groups RYGB+ (*n* = 8) and RYGB− (*n* = 6) were planned to be switched to an obesogenic high-fat diet enriched with sucrose (HF/S) (24, 25) 19 days before RYGB+/− (see Fig. 1).

### Implantation of Telemetry Devices

Right after initial weighing 28 days before RYGB+/−, all rats received radio telemetry devices [model TA10TA-F40; Data Sciences International (DSI), St. Paul, MN] for remote recording of body temperature and locomotor activity, which were implanted in the peritoneal cavity under isoflurane anesthesia after acclimatization (5% induction, 2% maintenance). A radio receiver (model RA1010; DSI) was mounted underneath each cage and attached via a BCM-100 consolidation matrix to a computerized data acquisition system (Dataquest IV, DSI). Measurement of body temperature and locomotor activity started at *preoperative day 7* and was continuously measured until the end of the experiment (*postoperative day 24*; sample interval 5 min, sample duration 10 s). To obtain a reference level of locomotor activity, the mean activity count from 1 wk before surgery was considered as 100% activity for that given animal. Activity counts were expressed as percentage of preoperative values, and group averages were calculated on transformed data (26). To distinguish circadian rhythmicity, body temperature and locomotor activity were divided into night phase (lights off) and day phase (lights on).

### Diets

The LF diet was standard chow [Altromin 1410), Altromin Spezialfutter GmbH & Co, Germany]. The high fat with sucrose (HF/S) diet was made in-house by using powdered standard chow (40.72% of total weight) of the same lot that was used for the LF condition in the present study, to which was added: lard (20%), soy oil (6.67%), sucrose (15%), arabic gum (5%), casein (10%), mineral mix (2.36%), and vitamin mix (1.55%; see Table 1). After thorough mixing, the HF/S dough was repelleted. For this purpose, the arabic gum [i.e., in a dose well below the level that was previously shown to induce changes in energy and nutrient balance (27)] was used to increase the toughness of the HF/S pellets. The minerals and vitamins were added to avoid deficiencies. To avoid fat oxidation, the HF/S was kept in a freezer (−20°C) and thawed before the exposure to the rats. The composition of the experimental diets is represented in Table 1. Food intake was monitored continuously from *preoperative day 7* until *postoperative day 24* by a computerized food intake monitoring system (TSE systems, Bad Homburg, Germany). A suspended food hopper, with spillage bin (sensor advanced, 259998-SEN/FED) was placed in the home cage of the rat by replacing the lid containing the food and water bottle with a custom-made lid in which the food hopper was fitted that was connected to the weighing system.

**Table 1.** Energy content of experimental diets

	Low-Fat Diet	High-Fat with Sucrose
Energy from fat, %	22	44.7
Energy from carbohydrates, %	50	35.7
Of which added sugar, %	-	45.5
Energy from proteins, %	28	19.6
Total energy, kJ/g	17.53	21.79

For the specifications of the standard chow see: https://altromin.com/products/standarddiets/rats/1410.

### Roux-en-Y Gastric Bypass Surgery

The RYGB surgery technique was taught in the laboratory of Prof. Dr. Lutz (Institute of Veterinary Physiology, Vetsuisse Faculty University of Zurich, Zurich, Switzerland) and was performed as described before (28). In short, following isoflurane anesthesia (5% induction, 2% maintenance), the abdomen was shaved and disinfected with surgical scrub and then a midline laparotomy was executed. In the RYGB+ surgery group, a 7-mm gastrotomy on the anterior wall was performed and ∼10% of the original size of the stomach was transected, the remaining gastric remnant was closed. The proximal jejunum was transected 15-cm distal to the pylorus to create the biliopancreatic limb. The common channel was made by transecting the ileum 25-cm proximal from the ileocecal valve where a 7-mm side-to-side jejuno-jejunostomy was performed between the biliopancreatic limb and the common channel. During sham surgery (RYGB−), a 7-mm gastrotomy on the anterior wall of the stomach and a 7-mm jejunotomy with subsequent closure at both locations were performed. Pre- and postsurgical care was provided with a subcutaneous injection of carprofen (5 mg/kg) at the start of the surgical procedure and 1 day after the surgical procedure. Animals were maintained on wet diet (LF or HF/S soaked in tap water provided in a jar placed on the floor of the cage, in addition to their food hoppers) for 5 days postsurgically, after which their respective preoperative solid diet was offered again.

### Euthanization

Rats were euthanized on *postoperative day 24*. Animals were placed in an isoflurane-filled chamber for sedation, followed by heart puncture. After careful excision, the weights of various fat depots within the abdominal cavity were isolated and weighted. Carcasses were deskinned, and weights of various subcutaneous fat depots were also isolated and weighted. The eviscerated carcasses, skin, and intestines of which the content was removed were dried in an oven at 65°C for 3 wk. After being dried and weighed, they were put in a petroleum-based soxhlet fat extractor (29) to dissolve the fat from these tissues. The fat weight from the skin was combined with weight of the preexcised subcutaneous fat depots to yield total subcutaneous fat (SC). Visceral (V) fat weight was calculated by combining weights from the preexcised depots in the abdominal cavity with fat extracted from the dried gut. Fat extracted from the dried carcasses was calculated as intramuscular (IM) fat. The remaining carcass weight was denoted as fat-free carcass (FFC) weight. Finally, total (T) fat content was calculated as the combined amount obtained from the various abovementioned fat fractions. Fat weight of organs (like liver, spleen, etc.) was not taken into account.

### Data Analysis

All data are expressed as means ± SE. Mortality due to surgical complications was: RYGB+ HF/S, *n* = 2 (25%) and RYGB+ LF, *n* = 6 (50%), and these animals were excluded from all analysis (χ^2^ test: n.s. between HF/S and LF groups). Three rats in the LF group and two rats in the HF/S group were found dead in their cage a few days after RYGB+ surgery. Postmortem analysis revealed gastrointestinal adhesions due to RYGB in all these rats. In addition, in the LF group, three rats were euthanized for reaching humane endpoints (like showing general sickness behavior, poor appearance, unresponsiveness, and adipsia). In only one of these, leakage was found in one of the anastomoses. Final group sizes were six rats per diet group that underwent RYGB+. In the RYGB− group, six rats were on the HF/S diet and eight rats were on the LF diet. Differences between groups were tested for significance using a two-factor ANOVA with post hoc Tukey’s pairwise multiple comparison with surgery (RYGB−, RYGB+) and diet (HF/S, LF) as factors. Where necessary, repeated-measure ANOVA was performed with surgery, diet, and time as separate factors. Regression analysis was performed to investigate which factors explained variation of weight loss following RYGB surgery. Particular care was taken not to inflate a regression model by checking for multicollinearity. *P* values <0.05 were considered statistically significant and statistics were calculated with IBM SPSS software 23.

## RESULTS

### Body Weight Loss and Energy Intake

**Figure 2.**
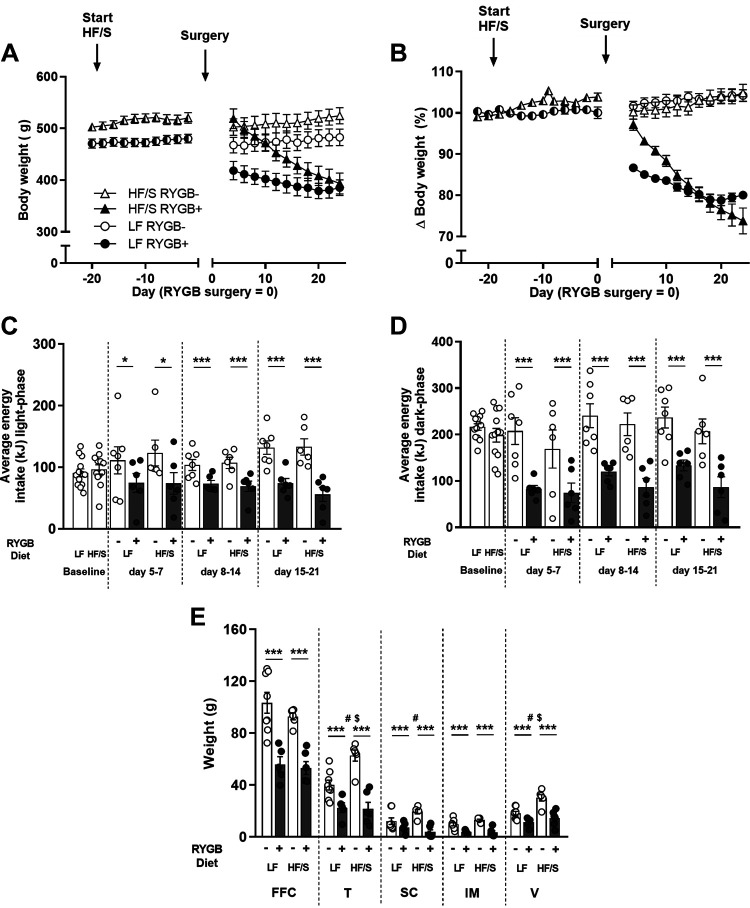
Effects of Roux-en-Y gastric bypass (RYGB+) and sham surgery (RYGB−) in rats on a high fat with sucrose (HF/S) or a low-fat (LF) diet on body weight in grams (*A*) and percentage (*B*), energy intake (*C*; light phase, *D*; dark phase), and components of body weight at the end of the experiment (*E*). Depicted components of body weight consisted of fat-free carcass (FFC) mass, total adipose tissue (T), subcutaneous adipose tissue (SC), intermuscular fat (IM), and visceral adipose tissue (V). Levels of significance are indicated as the following: *,***surgery effect; #diet effect; and $an interaction of surgery × diet (*P* < 0.05–0.001). Total group sizes: HF/S RYGB+ *n* = 6, LF RYGB+ *n* = 6, HF/S RYGB− *n* = 6, and LF RYGB− *n* = 8.

HF/S exposure before surgery caused rats to gain significantly more weight than the LF-feeding rats (resp. 19 ± 5 g vs. 6 ± 6 g g, *P* < 0.01), leading to elevated body weights in the HF/S group relative to the LF group (530 ± 15 g vs. 464 ± 11 g, *P* < 0.01, see Fig. 2*A*). Some variation in weight among groups already occurred between intraperitoneal implantation of telemetry devices (*day −28*) till diet switch (*day −19*) rendering the starting weight of the HF/S RYGB+ group a bit, albeit not significant, higher than the HF/S RYGB− group. After surgery, all RYGB+ rats lost weight, with the HF/S rats losing more weight than the LF rats (resp. 162 ± 16 vs. 96 ± 7 g; *P* < 0.01). In contrast, at the end of the experiment, the sham-surgery groups (RYGB−) had gained weight (LF 4 ± 5; HF/S 14 ± 5 g). Overall, RYGB− rats started to gain weight again after the first week postoperatively (Fig. 2*A*), whereas RYGB+ rats lost body weight until the end of the experiment (*postoperative day 24*). Throughout the postoperative period, time showed an interaction with surgery [*F*(20, 440 = 91.324; *P* < 0.0001] and with diet [*F*(20, 440 = 12.948; *P* < 0.0001] on body weight. Furthermore, there was an interaction between time × surgery × diet [*F*(2, 440) = 11.761; *P* < 0.0001], which could be explained to indicate that the HF/S RYGB+ rats had a steeper decline in body weight (30%) compared with the LF RYGB+ rats (20%, Fig. 2*B*). Overall, this weight loss resulted in significantly lower body weights [*F*(1,20) = 37.052; *P* < 0.0001] at the end of the experiment in the RYGB+ groups (HF/S RYGB+: 407 ± 20 g; LF RYGB+: 377 ± 16 g) compared with the RYGB− groups (HF/S RYGB−: 521 ± 16 g; LF RYGB−: 489 ± 21 g). Of note is the fact that the steeper weight loss trajectory in the HF/S RYGB+ rats kicked in a few days later compared with LF RYGB+ group, with the latter LF group losing quite some body weight already over the first 5 days, whereas this did not happen immediately in the HF/S group.

To see if variations in energy intake could explain RYGB+-induced weight loss, energy intake was measured continuously. During the baseline week, no significant differences were seen in energy intake between the different groups during the light and dark phases. After the first week following RYGB (during which moistened diet mash was given), rats that underwent RYGB+ consumed less than the RYGB− rats (Fig. 2, *C* and *D*), due to a surgery effect (post hoc Tukey’s test) that was seen both during the day [*week 1: F*(1,21) = 4.904, *P* < 0.05; *week 2*: *F*(1, 21) = 17.406, *P* < 0.0001; *week 3*: *F*(1, 21) = 37,019, *P* < 0.0001] and night [*week 1*: *F*(1,21) = 15.649, *P* < 0.0001; *week 2*: *F*(1, 21) = 30.576, *P* < 0.0001; *week 3*: *F*(1, 21) = 32.073, *P* < 0.0001] in all 3 wk after the surgical procedure (Fig. 2, *C* and *D*). No diet or surgery × diet effect on daily energy intake was seen during the day or night phases. When comparing the RYGB groups, however, total cumulative energy intake over the entire 3-wk postoperative period was significantly reduced in rats eating the HF/S diet (2302.3 ± 398.2 kJ) compared with the LF-fed rats (3158.5 ± 206.6; *P* = 0.027).

To determine the effect of RYGB on the weight of different body compartments, fat extraction was performed after euthanization (Fig. 2*E*). All body compartments showed a surgery effect, where RYGB+ animals had lower values compared with RYGB− animals [fat-free weight of the carcass (FFC); *F*(1, 21) = 46.474, *P* < 0.0001; total fat tissue (T); *F*(1, 21) = 47.683, *P* < 0.0001; subcutaneous fat (SC); *F*(1, 21) = 21.606, *P* < 0.0001; intermuscular fat (IM); *F*(1, 21) = 45.696, *P* < 0.0001; and visceral fat (V); *F*(1, 21) = 28.859, *P* < 0.0001]. In addition to a surgery effect, total [*F*(1, 21) =7.740, *P* < 0.05] and visceral fat tissue [*F* (1, 21) = 13.335, *P* < 0.01] showed a diet effect. Finally, total [*F*(1, 21) =5.559, *P* < 0.05], subcutaneous [*F*(1,21) = 6.2000, *P* < 0.05] and visceral fat [*F*(1,21) = 4.556, *P* < 0.05] showed a surgery × diet interaction effect. RYGB− HF/S rats had higher fat pad weights (T, SC, and V) compared with the RYGB− LF rats on *postoperative day 24*; however the RYGB+ HF/S rats had similar fat pad weights (T, SC, and V) compared with the RYGB+ LF rats.

### Diurnal Body Temperature and Physical Activity Patterns

**Figure 3.**
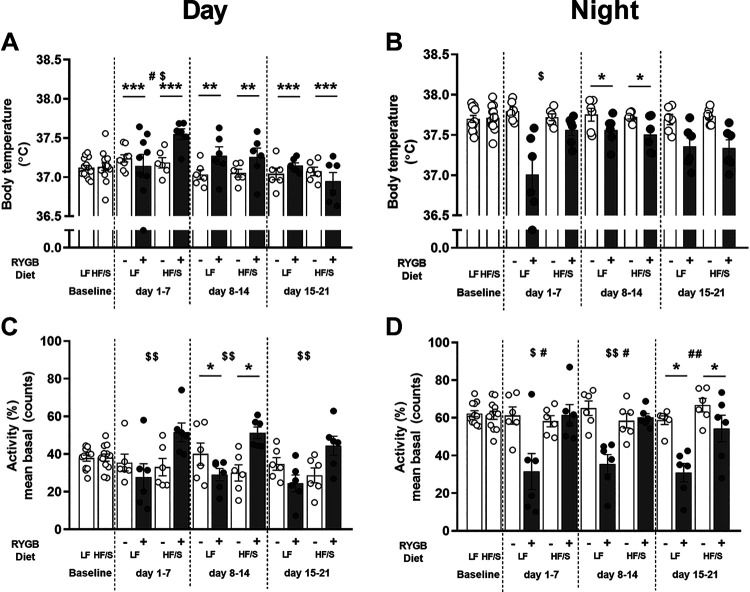
Effects of Roux-en-Y gastric bypass (RYGB+) and sham surgery (RYGB−) in rats on a high fat with sucrose (HF/S) or a low-fat (LF) diet on body temperature (*A*; lights on, *B*; lights off) and locomotor activity (%) of baseline (*C*; lights on, *D*; lights off). Levels of significance are indicated as the following: *,***surgery effect (*P* < 0.05-0.001); #, ##diet effect (*P* < 0.05-0.01); and $,$$an interaction of surgery × diet (*P* < 0.05-0.01). Total group sizes: HF/S RYGB+ *n* = 6, LF RYGB+ *n* = 6, HF/S RYGB− *n* = 6, and LF RYGB− *n* = 8.

Overall, RYGB surgery had an effect on both body temperature and locomotor activity rhythmicity (Supplemental Fig. S1) and diurnal patterns (Fig. 3) over the course of the experiment. During the first postoperative week, body temperature showed a surgery × diet interaction effect, with markedly lower levels found in the LF RYGB+ group [*F*(1,20) = 5.753, *P* < 0.05; Fig. 3*A*] during the light phase and higher levels in the HF/S RYGB+ group during the dark phase [*F*(1,20) = 8.010, *P* < 0.05; Fig. 3*B*]. During the entire postoperative period, body temperature during the dark phase was higher after RYGB surgery compared with the sham surgery [*week 1*: *F*(1,20) = 17.912, *P* < 0.001; *week 2*: *F*(1,20) = 8.537, *P* < 0.01; *week 3*: *F*(1,20) = 19.424, *P* < 0.001] but lower in the second postoperative week during the light phase [*F*(1,20) = 6.426, *P* < 0.05].

For locomotor activity (Fig. 3, *C* and *D*), surgery [light phase *week 2*: *F*(1,20) = 5.390, *P* < 0.05; dark phase *week 3*: *F*(1,20) = 7.774, *P* < 0.05] and diet [dark phase *week 1*: *F*(1,20) = 4.892, *P* < 0.05; *week 2: F*(1,20) = 6.299, *P* < 0.05; *week 3*: *F*(1,20) = 10.412, *P* < 0.01] showed significant effects. Furthermore, surgery showed an interaction effect with diet throughout the experiment. During the light phase, HF/S RYGB+ rats showed higher locomotor activity compared with the LF RYGB+ rats during all 3 wk after RYGB surgery [*week 1*: *F*(1,20) = 6.046, *P* < 0.05; *week 2*: *F*(1,20) = 15.265, *P* < 0.01; *week 3*: *F*(1,20) = 9.524, *P* < 0.01]. In contrast, LF RYGB+ rats showed reduced locomotor activity during the dark phase compared with the HF/S RYGB+ rats during all 3 wk after RYGB surgery [*week 1*: *F*(1,20) = 7.043, *P* < 0.05; *week 2*: *F*(1,20) = 17.095, *P* < 0.01]. With the RYGB groups alone, total (*P* = 0.02) and nighttime (*P* = 0.008) cumulative locomotor activity over the entire 3-wk postoperative period was significantly lower in the LF group relative to the HF/S group. Average daily body temperature over the entire 3-wk postoperative period was similar in the HF/S and LF diet groups.

### Regression Analysis

As mentioned earlier, we observed that RYGB-induced weight loss was larger in the HF/S-feeding rats than in the LF-feeding rats, which could potentially be explained by reduced energy intake and/or increased locomotor activity in the HF/S-feeding relative to LF-feeding rats. To further investigate this point, we performed regression analysis with “3-wk cumulative intake” and “3-wk cumulative locomotor activity” as independent factors, and “RYGB-induced weight loss” as its dependent factor. Although this model was significant (adjusted *R*^2^ = 0.519, *P* = 0.015), 3-wk cumulative energy intake (*P* = 0.012), but not 3-wk cumulative locomotor activity, contributed significantly to explaining the variation in weight loss. To further investigate whether and how the factor “diet” could explain the variation in weight loss, we reasoned that “diet” could either affect weight loss directly or that it did so by causing differences in body weight just before RYGB, which would also allow difference in the extent by which animals could lose weight after RYGB. Therefore, we performed two additional regressions with the same parameters as above, but with the first one supplemented with the independent factor “diet” (*R*^2^ = 0.79, *P* = 0.001) while the second one was supplemented with the independent factor “body weight before RYGB” (*R*^2^ = 0.87, *P* < 0.001). Adding “diet” and “body weight before RYGB” together was not allowed because they were dependent (i.e., according to multicollinearity testing). In both cases, the supplemented models better explained weight loss (with the second one being slightly superior to the first one), with all factors contributing significantly, except again for “3-wk cumulative locomotor activity.”

## DISCUSSION

In the present study, we consistently found that rats eating a Western-style diet high in fat and added sucrose (HF/S) exhibited increased body weight and augmented fat storage compared with rats eating a healthy low-fat (LF) laboratory diet (24, 25, 30), which was successfully reversed by RYGB surgery. RYGB-induced weight loss (both absolute and as % weight loss) was clearly higher in the HF/S-fed rats relative to the LF-eating rats, yielding roughly the same body weights 3 wk following RYGB. In contrast, the sham-operated animals (both HF/S and LF) showed stable body weight during the whole postoperative period, indicating negligible effect of opening the abdominal cavity without the RYGB procedure. Detailed carcass analysis at *postoperative day 24* showed an interaction effect between diet and surgery on total, subcutaneous, and visceral fat tissue, meaning that HF/S RYGB+ animals lost significantly more fat tissue compared with the LF RYGB+ animals. Consequently, the weight of the different body compartments (fat-free carcass and different fat tissues) was similar in both diet groups 3 wk after RYGB+ surgery, whereas in the control RYGB− groups, these were elevated in the HF/S versus the LF rats. The augmented RYGB-induced weight loss in HF/S- relative to LF-fed rats was found without differences in energy intake during the light and dark phases over the three separate consecutive weeks. However, when measured cumulatively over the entire 3-wk period, the RYGB rats eating an HF/S diet rats consumed significantly less energy compared with the RYGB rats eating an LF diet, which could have contributed to the augmented weight loss in the RYGB HF/S-fed rats. At this point, we do not know whether the HF/S diet was tolerated or preferred less, which should be tested in future experiments. In addition, it would be of interest to study floor effects of body (fat) weight in a longer-term study on the interactions between diet and surgery in the current setup. Finally, RYGB surgery may also have augmented malabsorption in the HF/S group as a certain degree of reduction of fat digestibility has been reported following RYGB in rats and mice (31–34) but less so for carbohydrate and protein digestibility (35). However, the extent to which fecal fat loss affects weight loss is still under debate (32, 36, 37).

Before RYGB, consumption of the HF/S diet leading to increased weight relative to the LF diet condition, presumably yielded more available metabolic substrates (38), which could potentially have contributed to the phenomenon that rats eating the HF/S diet have an increased locomotor activity relative to rats eating the LF diet following RYGB. Fueling locomotor activity (39) could be energy costly and might have raised energy expenditure in the rats eating an HF/S relative to those eating an LF diet (40), which in turn could have led to additional weight loss that was not explained by energy intake reduction per se. The extent to which the variation in body weight loss following RYGB was explained by 3-wk cumulative locomotor activity and 3-wk cumulative energy intake was further investigated in a regression analysis. Although both factors collectively explained 51% of the variation in body weight loss, only 3-wk energy intake contributed significantly to the variation in RYGB-induced weight loss. This model was further improved by the addition of body weight just before RYGB, finally explaining 87% of the variation in RYGB-induced weight loss, but again without a significant contribution of locomotor activity. The fact that the amount of weight loss following RYGB also significantly depended on the starting weight just before the surgical procedure is consistent with findings by others (41, 42). Replacing the factor “body weight just before RYGB” by “diet” yielded roughly the same outcome in the regression, suggesting that the effect of diet was perhaps mostly mediated via its effect on presurgery weight and body fat content. However, we cannot dissociate to what extent diet type had a direct effect on RYGB-induced weight loss, beyond its capacity to differentiate body weight in the period before RYGB (with HF/S-feeding rats being significantly heavier than LF-feeding rats). For that purpose, it would be of interest for future studies to investigate the effect of diet switches (e.g., from HF/S back to LF and vice versa) around the time of RYGB, to investigate direct versus indirect effects of diet type on RYGB-induced weight loss.

It is noteworthy that animals on the HF/S diet only showed an increase in locomotor activity during the light phase after RYGB surgery. In contrast, animals on the LF diet showed a decrease in locomotor activity. An increased locomotor activity during the light phase could be expected when animals that underwent RYGB eat smaller meals, but with a higher frequency spread out during the whole 24 h as shown by Zheng et al. (43). Whether such an effect is related to the nutritional value or macronutrient composition of the diet, release and responsiveness to gut hormones, or whether it is related to specific brain mechanisms needs to be further investigated (e.g., by experiments using diet switches as proposed above, and the use of running wheels to investigate more specifically the role of locomotor activity in energy balance following RYGB). Such experimentation should optimally be performed using purified diets, because in the present study, we cannot rule out the fact that certain unspecific ingredients other than fat and sucrose in the two different diets could have altered the behavioral and/or physiometabolic responses to RYGB in the two diet groups (44).

Despite the fact that the weight loss trajectory was larger in the HF/S-feeding rats relative to the LF feeding rats, it appeared that HF/S-feeding rats had a more rapid recovery from RYGB than the LF-feeding ones, judged on the basis of the less severely reduced circadian amplitudes of body temperature and locomotor activity in the HF/S rats versus the LF rats. The pattern and amplitude of circadian rhythms of these parameters can be used to indicate malaise induced by the surgery during the first week after RYGB (23, 45). The observation that the weight loss trajectory in the LF RYGB+ rats in the present study appeared to kick in a few days earlier compared with HF/S RYGB+ group might also be related to increased malaise in the LF group. Indeed, some rats in the LF RYGB+ group had diarrhea during the first few days after surgery, but this was unfortunately not quantified. It might be argued that the increased intra-abdominal fat content before RYGB+ surgery of the HF/S group relative to the LF group rats could have diffused some of the immunological responses that are associated with gastrointestinal surgery (23, 46). In line with this reasoning is the finding by Komegae et al. (47), who also reported a much reduced hypothermic response following injection of the endotoxin LPS in diet-induced obese rats versus lean rats on a standard diet. A bias toward hypothermia resistance might provide the basis for the so-called obesity paradox, according to which obesity may improve the outcome of acute infections under at least some circumstances, despite being a risk factor for so many chronic diseases (48).

### Perspectives and Significance

Our results show that rats undergoing RYGB surgery exhibited weight loss mediated by food intake reduction that was more profound in the HF/S-fed obese rats than the LF-fed rats. The degree of weight loss in a regression model was explained, besides energy intake reduction, also by body weight just before RYGB, and this relation was not influenced by factors related to locomotor activity. An intriguing observation in our study was that the short-term survival and recovery following RYGB surgery appeared to be improved in HF/S-fed rats relative to LF rats, suggesting that the more profound weight loss in the HF/S group was not due to unspecific effects (such as malaise). Unraveling the neurobiological mechanisms by which stored fat and/or diet type independently or in combination exert these effects may be of clinical importance and deserves further investigation.

## DATA AVAILABILITY

Source data for this study are openly available at https://doi.org/10.34894/S0X3TY.

## SUPPLEMENTAL DATA

10.34894/S0X3TY Supplemental Fig. S1: https://doi.org/10.34894/S0X3TY.

## DISCLAIMERS

The content is solely the authors’ responsibility.

## DISCLOSURES

No conflicts of interest, financial or otherwise, are declared by the authors.

## AUTHOR CONTRIBUTIONS

C.W.H., E.S., T.A.L., A.P.v.B., and G.v.D. conceived and designed research; C.W.H. and J.E.B. performed experiments; C.W.H., J.E.B., and G.v.D. analyzed data; C.W.H., E.S., C.N.B., T.A.L., M.E., A.P.v.B., and G.v.D. interpreted results of experiments; C.W.H. prepared figures; C.W.H. and E.S. drafted manuscript; C.W.H., J.E.B., C.N.B., T.A.L., M.E., A.P.v.B., C.N., and G.v.D. edited and revised manuscript; C.W.H., J.E.B., C.N.B., T.A.L., M.E., A.P.v.B., C.N., and G.v.D. approved final version of manuscript.
